# Overgeneralization of conditioned fear in patients with social anxiety disorder

**DOI:** 10.3389/fpsyt.2024.1415135

**Published:** 2024-08-23

**Authors:** YoonJi Irene Lee, Dasom Lee, Haena Kim, Min Joo Kim, Heekyoung Jeong, Dongseob Kim, Evelyn Glotzbach-Schoon, Soo-Hee Choi

**Affiliations:** ^1^ Department of Psychiatry, Seoul National University Hospital, Seoul, Republic of Korea; ^2^ Department of Psychological and Brain Sciences, Texas A&M University, College Station, TX, United States; ^3^ Department of Psychiatry, Seoul National University College of Medicine and Institute of Human Behavioral Medicine, Seoul National University-Medical Research Center (SNU-MRC), Seoul, Republic of Korea

**Keywords:** social anxiety disorder, fear conditioning, overgeneralization, threat appraisal, arousal

## Abstract

**Introduction:**

While abnormal responses to threat, including overgeneralization to conditioned fear, have been postulated to play a critical role in pathological anxiety, the relevance of previous findings to social anxiety disorder (SAD) is unclear. We investigated conditioned and generalized fear responses in patients with SAD using socially relevant stimuli.

**Methods:**

A total of 26 patients with SAD and 25 healthy controls participated in a fear conditioning and generalization paradigm consisting of two neutral faces as conditioned stimuli (CS+ or CS−) and an angry face with contemptuous comments as unconditioned stimuli. Eight morphed faces of two conditioned stimuli in each continuum were given to test generalization. Behavioral data and physiological responses were acquired.

**Results:**

Successful conditioning was observed in the risk ratings for both groups, while only a marginal indication of conditioning was noted in physiological measures. During the generalization phase, patients rated the risk higher than CS− when the stimuli close to CS− contained a portion of CS+ features. Larger skin conductance responses to this stimulus were linked to higher fear of negative evaluation. In addition, patients spent a longer time evaluating safe and ambiguous stimuli than healthy controls and exhibited consistently high levels of subjective arousal.

**Discussion:**

Taken together, our findings suggest that SAD patients may exhibit a tendency towards overgeneralization of fear responses and show distinct patterns in processing generalized threat stimuli compared to healthy controls. Even though overgeneralization was not evident in physiological measures, it is necessary to consider this behavioral characteristic in the clinical management of patients with SAD.

## 1 Introduction

Social anxiety disorder (SAD) is one of the most common mental disorders, with 13% life prevalence in the United States, 8% in Canada, and 8.4% in Australia (1–3). SAD is characterized by an intense and persistent fear of being judged, criticized, or embarrassed in social situations, leading to extreme distress and/or avoidance of social situations (4). A diagnosis of SAD is associated with an increased risk of developing other mental disorders such as mood disorders, other anxiety disorders, substance use disorders, and independently predicts suicidal ideation, attempts, and completion (5–7). The co-occurring mental health challenges linked to SAD not only pose significant individual burdens but also contribute to a broader societal impact, increasing indirect costs, such as reduced workplace productivity (8).

Fear conditioning has been widely employed as a laboratory model to investigate the development and maintenance of fear and anxiety (9–13). Classical fear conditioning represents the learning process by which a neutral stimulus, through repetitive association with an unconditioned aversive stimulus (US), comes to induce fear responses and consequently becomes a conditioned stimulus (CS+). Individuals with anxiety disorders have been reported to exhibit maladaptive characteristics in distinct mechanisms of fear conditioning: 1) impaired fear extinction and 2) overgeneralization of conditioned fear (11–14). Fear extinction typically involves a decrease in conditioned fear response after repeated exposure to the CS+ in the absence of the US. However, findings from previous meta-analyses have shown that during extinction trials, patients with anxiety-related disorders tend to display heightened autonomic fear responses to the CS+ that is no longer predictive of the US (9, 10). Furthermore, compared to controls, patients demonstrate increased differentiation between the CS+ and the CS− (the safe stimulus that was never paired with the US) during extinction (9, 10). These results suggest that conditioned fear responses to the CS+ are more resistant to extinction in patients compared to controls. In addition to impaired fear extinction, patients with anxiety disorders often exhibit maladaptive fear generalization, where conditioned fear responses extend beyond the original CS+ to other stimuli resembling it. While fear generalization serves as an adaptive mechanism that protects an individual from potential harm, it becomes maladaptive when fear extends to a broader range of harmless stimuli. Emerging evidence indicates heightened fear generalization in anxiety disorders, as measured by stronger conditioned fear responses to generalization stimuli resembling the CS+ but never paired with the US, compared to healthy controls (14).

Conceptually, fear generalization can play a crucial role not only in the acquisition of fear but also in the maintenance of SAD (15). For instance, an individual may develop fear after experiencing the humiliation of being criticized during a public speech in a specific location. The fear response stemming from this negative feedback may generalize to different environments or other social situations, subsequently leading to avoidance behavior and, thus, impeding safety learning. Difficulties in distinguishing between danger and safety cues have been experimentally demonstrated in previous studies in SAD (16–18). However, given that anxiety-evoking stimuli outside laboratory setting are generally more uncertain and vague, a fear generalization paradigm using ambiguous generalization stimuli is likely to better reflect clinical anxiety compared to tests involving simple discrimination between CS+ and CS− (14). In addition, anxiety-inducing stimuli vary across different psychopathological dysfunctions. When applying the fear conditioning paradigm to social anxiety subjects, researchers have found that socially relevant stimuli such as emotional facial expressions and verbal feedback induce greater fear responses in patients (19) and highly socially anxious individuals (20). Such patterns, however, have not been observed in experiments using other nonspecific aversive stimuli such as odor or painful pressure (18, 21). This underscores the significance of incorporating disorder-relevant stimuli in the fear conditioning paradigm.

To date, few studies have investigated the fear generalization in SAD. Ahrens et al. employed two faces as CS+ and CS−, along with a loud scream and a fearful face as the US (17). They assessed generalization by presenting both CSs and ambiguous generalization stimuli comprising four morphs of the two faces. The researchers concluded that there is no solid and robust evidence of overgeneralization in SAD and suggested that, while socially relevant, the screaming sound employed as the US might not have been disorder-specific enough to elicit an overgeneralization pattern in patients (17). Given that the core fear of SAD is related to social or performance situations wherein negative judgment is anticipated (4), stimuli such as angry faces and contemptuous comments may be more appropriate as US (17, 19, 20). Incorporating these disorder-relevant stimuli into the fear generalization paradigm could offer a more detailed and clinically relevant understanding of social anxiety.

The main objective of this study was to investigate whether an ecologically enhanced fear conditioning paradigm using disorder-specific stimuli (i.e., negative evaluation) can elicit a maladaptive pattern of overgeneralization in social anxiety. To this aim, we employed a social conditioning paradigm (19) using pictures of male/female faces as the CSs and social anxiety-related aversive stimulus (contemptuous auditory comment) as the US. In the paradigm, the generalization phase following fear acquisition involved generalization stimuli (GS), which were eight morphed faces along a continuum between CS+ and CS−, none of which were paired with the US. Fear responses were assessed by behavioral (risk rating and reaction times), physiological (skin conductance and heart rate), and fear-potentiated startle (electromyography, EMG) responses. Based on the generalization paradigm, we anticipated that, as the presented GSs decrease in resemblance to CS+, subjects would exhibit a decrease in risk ratings, skin conductance, and startle EMG but an increase in heart rate (due to more pronounced heart rate deceleration, i.e., fear bradycardia, to stimuli closer to CS+). As an indicator of stimulus discrimination, reaction times were expected to be slower for ambiguous GSs resembling both CS+ and CS− to some extent. Based on previous findings that socially anxious individuals have difficulties in discriminating threat and safety cues (16–20), we hypothesized that patients with SAD, compared to controls, would generalize their conditioned fear responses to a wider range of GSs. Specifically, this would manifest as heightened fear responses to GSs closer to CS− than to CS+ in patients (but not in controls).

## 2 Materials and methods

### 2.1 Participants and measurements

A total of 28 patients with SAD and 39 healthy controls were recruited from the psychiatric outpatient clinic and the community through an advertisement. One patient and two controls dropped out of the study, as the loud acoustic startle probe was intolerable for them. Physiological data acquired from one patient was missing. A total 12 controls were excluded due to high levels of social anxiety and/or depressive symptoms. These participants had an average Liebowitz Social Anxiety Scale score of 42.9 (standard deviation=10.9), which was higher than the mean of 18.0 (standard deviation=9.6) for the included controls. However, they volunteered as controls, did not report social anxiety symptoms, and did not meet the full diagnostic criteria for SAD. Ultimately, 26 patients with SAD and 25 controls were included in the statistical analyses. As presented in **Table 1**, the demographic variables, including age, sex, and education, did not differ significantly between the two groups.

**Table 1:** Demographic and descriptive characteristics of the participants.

Variable	SAD (*N* = 26)		Controls (*N* = 25)		*χ^2^* or *t*
	*N*	%	*N*	%	
Male	11	42.3	10	40	0.028
	Mean	SD	Mean	SD	
Age, in years	24.7	3.1	24.0	2.9	1.367
Educational level, in years	15.1	1.7	15.9	1.8	1.541
Liebowitz Social Anxiety Scale, 0–144	77.2	19.3	18.0	9.6	13.944*
Social Interaction Anxiety Scale, 0–80	53.2	14.8	14.2	7.8	11.871*
Social Phobia Scale, 0–80	37.7	17.7	4.2	3.7	9.428*
Brief Fear of Negative Evaluation Scale, 12–60	45.6	11.0	26.9	8.3	6.874*
Hamilton Anxiety Scale,^a^ 0–56	27.0	10.4	11.0	9.4	5.394*
Beck Anxiety Inventory,^b^ 0–63	17.5	11.8	2.6	3.1	5.983*
State-Trait Anxiety Inventory—State,^b^ 20–80	51.2	8.8	31.7	8.3	7.719*
State-Trait Anxiety Inventory—Trait,^b^ 20–80	58.0	11.1	34.0	9.1	8.017*
Beck Depression Inventory, 0–63	18.9	11.3	4.4	5.3	5.899*
Montgomery–Åsberg Depression Rating Scale,^a^ 0–60	15.3	8.3	1.8	2.5	7.281*
Sheehan Disability Scale,^a^ 0–30	17.3	5.9	4.1	4.6	8.168*

SAD, social anxiety disorder.

a Data from four controls and four patients.

b Three controls and two patients were missing.

*p <.001.

Patients were diagnosed with SAD when they met the full criteria for SAD according to the Diagnostic and Statistical Manual of Mental Disorders Fifth Edition through an intensive and open-ended clinical interview with a psychiatrist. When SAD was the primary diagnosis, patients who had related depressive disorder were also included. Through an additional structured interview, specifically the Mini-International Neuropsychiatric Interview (22), a psychologist assessed patients’ comorbidities and excluded control participants with past or current psychiatric disorders. Exclusion criteria for both groups included a past or current history of neurological disorders, pregnancy, or a past or current diagnosis of psychosis. The numbers of patients with past or current comorbid psychiatric disorders were as follows: major depression (n = 20), panic disorder (n = 3), agoraphobia (n = 7), general anxiety disorder (n = 6), obsessive–compulsive disorder (n = 1), eating disorder (n = 3), and premenstrual dysphoric disorder (n = 8).

To measure the severity of clinical symptoms, participants completed self-reported questionnaires, including the Liebowitz Social Anxiety Scale (LSAS) (23), the Social Interaction Anxiety Scale (SIAS) and the Social Phobia Scale (SPS) (24), a brief version of the Fear of Negative Evaluation scale (B-FNE) (25), the State-Trait Anxiety Inventory (STAI) (26), the Beck Anxiety Inventory (BAI) (27), and the Beck Depression Inventory (BDI) (28). Participants were also assessed using the Hamilton Anxiety Scale (HAS) (29), Montgomery–Åsberg Depression Rating Scale (MADRS) (30), and Sheehan Disability Scale (31). Patients showed overall higher clinical symptom scores than the controls, including the scores for general anxiety (STAI, BAI, and HAS) and depressed mood (BDI and MADRS), and social anxiety severity (LSAS, SIAS, SPS, and B-FNE) (**Table 1**).

The study protocol was approved by the Institutional Review Board of Seoul National University Hospital and was conducted in accordance with the Declaration of Helsinki. Written informed consent was obtained from all participants.

### 2.2 Stimulus

Two pictures of actors with neutral facial expressions were selected from the Korean Facial Expressions of Emotion (32) as the CS. An audiovisual stimulus comprising an angry facial expression of a threat cue (CS+) paired with a simultaneously presented contemptuous comment (e.g., “you are good for nothing!”) served as the US. The other CS face was included as a safety cue (CS−) and was associated with the neutral content (e.g., “banana is yellow”). Details about the selection of the US are described in **Supplementary Material**. Given the clinical findings that the level of social anxiety increases when dealing with the opposite sex (33), the participants were conditioned with pictures of the opposite sex. Female participants were presented with CS featuring male faces and US with a male voice. Conversely, male participants were exposed to CS featuring female faces and US with a female voice. The order of the stimuli was pseudo-randomized, and the allocation of which actor was assigned as being the CS+ was also balanced out across the participants.

**Figure 1:**
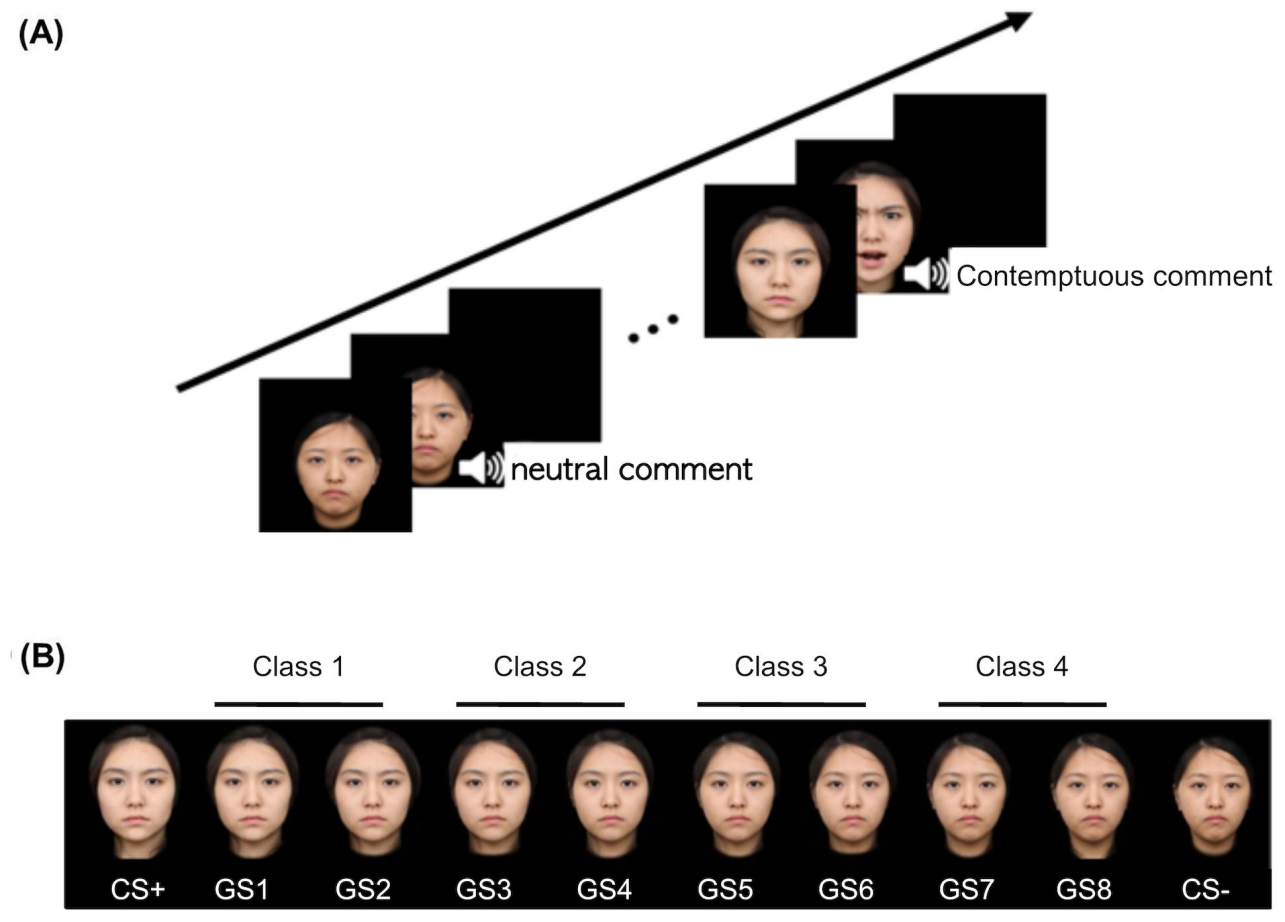
An example of conditioning and generalization stimuli. **(A)** A safe cue (CS−) was paired only with a neutral comment and neutral face, while a threat cue (CS+) was paired with a contemptuous comment and an angry face. **(B)** Generalization stimuli comprise eight morphed faces of two conditioning stimuli, which were collapsed into four different classes such that Class 1 is the closest to the CS+ and Class 4 to the CS−. Each item of GSs differs from the other by 12.5%. CS, conditioned stimuli; GS, generalized stimuli.

Eight morphed faces of the CS faces in each continuum were given to test generalization (GS); GS1 refers to the one closest to CS+, and GS8 was closest to CS–. Then, we grouped the two adjacent stimuli as one class, i.e., GS1 and GS2 as Class 1 (C1), GS3 and GS4 as C2, and so forth. Hence, C1 was the most similar to CS+, and C4 showed the greatest difference with respect to CS+ (**Figure 1**).

The startle probe, a burst of white noise (103 dB, 50 ms) was given binaurally through headphones (34).

### 2.3 Design and procedure

The experiment paradigm comprises the habituation of the startle sound, pre-acquisition, acquisition, and generalization phases. In the habituation phase, four trials of the white noise were presented with a blank screen. This phase was introduced to facilitate participants’ adaptation to the startle probe, and no specific instructions were provided. After the habituation phase, the pre-acquisition, acquisition, and generalization phases were presented sequentially. Throughout each phase, participants were instructed to provide risk ratings indicating the likelihood of the US occurring while observing the presented facial stimuli. At the end of each phase, participants were directed to rate the valence, arousal, and threat associated with the presented facial stimuli. Detailed explanations regarding the ratings will be provided below. The pre-acquisition phase consisted of six CS+ and six CS− with no subsequent US presentation. During the acquisition phase, 12 of each of the CS faces were presented with 83% of the reinforcement schedule, i.e., 10 of each CS trial were followed by either a neutral comment with a neutral facial expression (CS−) or a contemptuous comment with an angry facial expression (CS+). Similarly, in the generalization phase, a total of 12 trials of each CS and six trials per GSs were given. To prevent early extinction, 50% of the reinforcement schedule was given during the generalization phase. All CSs and GSs over a black background were presented for 6 s on the center of a screen. The US, either neutral or contemptuous, followed immediately for 3 s. A blank screen with a black background was presented for either 9 s or 11 s as an interval inter-trial to minimize the expectancy effect. The startle probe was given in half of the trials at either 4 s or 5 s after the onset of the CS.

Participants’ subjective risk ratings and reaction times (RTs) for these risk ratings were measured as behavior indices. During half of the CS and GS trials, the risk rating scale appeared at the bottom of the screen 3 s after stimulus onset. The scale ranged from 1 (not at all) to 3 (highly probable). Participants were instructed to rate the likelihood of hearing a contemptuous comment using three buttons on a numeric keyboard. Subjects were told to respond as quickly as possible to each stimulus. At the end of each pre-acquisition, acquisition, and generalization phase, participants were instructed to rate the valence, arousal, and threat for CS+ and CS−. Each stimulus was presented on the screen, followed by three 9-point Likert scales, each reflecting levels of valence (“How negative or positive was the stimulus?,” 1 = negative, 9 = positive), arousal (“How arousing was the stimulus?,” 1 = low, 9 = high), and threat (“How threatening was the stimulus?,” 1 = not at all, 9 = threatening). Participants were instructed to respond using the nine buttons on a numeric keyboard.

The paradigm was delivered by Presentation® software (Neurobehavioral Systems Inc., Berkeley, CA, USA) on a monitor at approximately 60 cm.

### 2.4 Physiological data acquisition and analysis

The participants underwent the study individually in a temperature- and humidity-controlled room (23°C ± 3°C and 40% ± 5%, respectively). Skin conductance level (SCL), heart rate (HR) as a physiological response, and EMG as the startle response were acquired with an MP-150 system (BIOPAC Systems Inc., Goleta, CA, USA) at a sample rate of 1,000 kHz and analyzed using AcqKnowledge software (BIOPAC Systems Inc.). For all measurements, data with less than two valid responses per stimuli for each phase were excluded (35).

Skin conductance level was recorded using two Ag/AgCl electrodes filled with an isotonic electrode gel from the distal phalanges of the second and third digits of the non-dominant hand. The acquired skin conductance recording was filtered by a 50-Hz notch filter and a 1-Hz cutoff filter. Responses lower than 0.02 μS were filtered out. Based on previous studies that applied a range correction to SCL to account for individual differences (36–39), the SCL values were calculated by subtracting the minimum SCL (within 0–2 s before the stimulus picture onset) from the stimulus-specific onset SCL and then dividing by the difference between the maximum [within 1–6 s following the stimulus picture onset (40)] and the minimum SCL. The results of the analyses using different time windows are presented in **Supplementary Material**.

HR was measured using a photoplethysmography transducer at 50 Hz from the fourth digits of the non-dominant hand. First, the estimated beats per minute for each stimulus (1–6 s from the stimulus picture onset) and the baseline HR of 2 s before each onset was automatically driven by the AcqKnowledge software and then averaged out. Then, we deducted the baseline HR estimate from the averaged HR.

Eyeblink magnitude to the startle was measured using EMG from the orbicularis oculi with two Ag/AgCl electrodes (diameter, 4 mm) placed on the lower eyelids centrally located in line with the pupil and under the lateral canthus of the left eye. In addition, the isolated ground electrode was placed at the mastoid. Impedance was kept below 5 kΩ. The acquired signal was amplified and filtered with a 50-Hz notch filter and band-pass at 28–500 Hz. Then, it was rectified and smoothed with a 1,000-Hz sample rate. Baseline correction was performed for over 50 ms before the startle onset. The peak EMG per stimuli was calculated with a time window of 50–200 ms after the startle onset. Artifact rejection was completed manually with an exclusion criterion of responses with baseline shifts above or below 5 μV. Trial-irrelevant movements occurring at the end of the session were eliminated, and the percentage fell within the range of 3%–5%. No trials were excluded due to movement artifacts. Within-subject T-score standardization was applied to the EMG magnitudes across all phases.

### 2.5 Statistical analysis

Statistical analyses were conducted using SPSS (version 26; SPSS Inc., Chicago, IL, USA) and R (R Foundation for Statistical Computing, Vienna, Austria, 2012). In pre-acquisition, we applied 2 (Group: controls, SAD) × 2 (Condition: CS+, CS−) rmANOVA. To assess the acquisition of conditioning over time, the acquisition phase was analyzed by dividing it into early and late phases. Each phase was defined as the first (6 CS+ and 6 CS−) and the second halves (6 CS+ and 6 CS−) of the experiment. Then, we carried out 2 (Group: controls, SAD) × 2 (Condition: CS+, CS−) × 2 (Phase: early, late) rmANOVA. In the generalization phase, we carried out 2 (Group: controls, SAD) × 6 (Stimulus: CS+, C1, C2, C3, C4, and CS−) rmANOVA. Dependent variables were risk rating, RT, SCL, HR, and startle EMG. No SCL data were available for CS− during the late acquisition phase for one participant in the CON group. Additionally, no startle EMG data were available for CS+ during the early acquisition phase for another participant in the CON group. As a result, each participant was excluded from the SCL and startle EMG analysis of the acquisition phase, respectively, but was included in all other analyses.

To test across-phase changes in subjective ratings and physiological measures for CS+ and CS−, 2 (Group: controls, SAD) × 2 (Time: acquisition, generalization) × 2 (Condition: CS+, CS−) rmANOVA was conducted. Dependent variables include subjective ratings of valence, arousal, and threat and physiological outcomes such as SCL, HR, and startle EMG. Lastly, we investigated whether these behavioral and physiological outcomes are correlated with social anxiety symptoms such as LSAS, SIAS, SPS, and FNE. Pearson correlation analysis was conducted followed by partial correlation analysis to control for the effect of general anxiety, with trait anxiety serving as a control variable.

For analyses of variance, the Greenhouse–Geisser correction (ϵ) was applied when the assumption of sphericity was violated. Analyses incorporating BDI and BAI as covariates were also conducted, and the results can be found in **Supplementary Material**. The Benjamini–Hochberg (B–H) correction was used to correct the alpha level of multiple comparisons for *post-hoc t*-tests (30) except for correlation analysis. A significance level for the *p*-value of 0.05 was applied across the analyses, and the effect sizes were reported using partial eta-squared (*η^2^_p_*).

Sample size was determined by power calculations for repeated measures ANOVA, using an effect size of 0.77 from a previous fear conditioning study in social anxiety disorder (17). With an alpha of 0.05 and a statistical power of 0.95, the sample size of 20 for each group is required.

## 3 Results

### 3.1 Pre-acquisition phase

The rmANOVA results with two factors (i.e., Group as a between-subject factor and Condition as a within-subject factor) revealed no significant main or interaction effects on risk rating (all, *p* >.05). For RT, the main effect of Condition was the only significant result [*F*(1,44) = 4.087, *p* =.049, *η^2^_p_* =.085]; RT of CS− was longer than that of CS+. Results regarding physiological outcomes indicated that there was no significant main or interaction effect on SCL, HR, or startle EMG (all, *p* >.05).

### 3.2 Acquisition phase

For risk rating, a significant Condition × Time interaction was observed [*F*(1,45) = 19.375, *p* <.001, *η^2^_p_* =.301] (**Table 2**). *Post-hoc t*-tests indicated that the risk rating for CS+ increased significantly from 2.34 to 2.71 over time (*t* = −5.429, *p* <.001), whereas there was no significant change for CS− (*t* = 1.510, *p* =.130) (early: 1.23, late: 1.12). Further examination of changes in risk rating for CS− within each group revealed a significant decrease from 1.30 to 1.02 in controls (*t* = 2.658, *p* = .015). However, risk rating for CS− did not change in SAD (*t* = −0.334, *p* =.741) (early: 1.18, late: 1.21), indicating that SAD did not adjust their threat evaluation for CS− even after repeated learning that CS− is not associated with US. The main effects of Condition [*F*(1,45) = 375.908, *p* <.001, *η^2^_p_* =.893] and Time [*F*(1,45) = 7.027, *p* =.011, *η^2^_p_* =.135] were significant. No other significant main or interaction effect was found.

**Table 2:** Descriptive statistics for behavioral and physiological outcomes.

Variable		ACQ-early		ACQ-late		Generalization					
		CS+	CS−	CS+	CS−	CS+	C1	C2	C3	C4	CS−
Risk rating	Controls	2.32 (0.51)	1.30 (0.50)	2.68 (0.52)	1.02 (0.07)	2.42 (0.61)	2.34 (0.61)	1.75 (0.58)	1.16 (0.23)	1.05 (0.14)	1.11 (0.25)
	SAD	2.37 (0.50)	1.18 (0.28)	2.73 (0.43)	1.21 (0.37)	2.63 (0.45)	2.56 (0.51)	1.94 (0.65)	1.36 (0.31)	1.10 (0.27)	1.15 (0.28)
RT	Controls	137.27 (39.09)	122.77 (34.44)	123.71 (46.46)	110.09 (35.34)	120.11 (41.72)	122.96 (40.02)	124.79 (35.74)	122.67 (26.81)	111.52 (36.90)	109.03 (36.45)
	SAD	148.01 (54.69)	139.06 (45.96)	136.59 (53.67)	131.58 (45.64)	128.58 (37.37)	143.68 (49.40)	160.7 (42.03)	141.88 (39.63)	138.08 (38.78)	142.39 (42.06)
SCL	Controls	0.40 (0.37)	0.27 (0.44)	0.48 (0.21)	0.41 (0.20)	0.37 (0.17)	0.30 (0.19)	0.32 (0.22)	0.44 (0.16)	0.33 (0.16)	0.37 (0.15)
	SAD	0.43 (0.26)	0.33 (0.28)	0.39 (0.18)	0.42 (0.22)	0.40 (0.17)	0.37 (0.30)	0.49 (0.31)	0.38 (0.20)	0.41 (0.25)	0.45 (018)
HR	Controls	−5.47 (10.26)	−5.19 (10.45)	−3.79 (11.58)	−2.24 (6.54)	−2.29 (6.85)	−1.03 (7.02)	−1.77 (7.41)	−0.76 (6.13)	−1.25 (6.79)	−1.78 (6.65)
	SAD	−2.54 (7.76)	−2.04 (3.44)	−3.49 (10.98)	−2.99 (8.15)	−1.63 (8.76)	−1.96 (7.71)	−2.05 (8.13)	−1.14 (8.25)	−1.42 (7.66)	−1.97 (8.19)
Startle EMG	Controls	65.60 (8.39)	64.50 (9.85)	66.09 (8.28)	64.57 (9.73)	60.2 (7.91)	59.35 (6.09)	58.47 (6.21)	59.43 (6.47)	60.64 (7.10)	60.29 (5.35)
	SAD	66.88 (7.20)	64.67 (6.44)	66.57 (7.22)	64.33 (6.40)	61.64 (5.10)	60.4 (5.47)	60.89 (5.87)	61.34 (5.74)	62.28 (7.18)	60.97 (5.69)

Data are shown as mean (standard deviation).

SAD, social anxiety disorder; RT, reaction time; SCL, skin conductance level; HR, heart rate; EMG, electromyography; CS+, threat cue; CS-, safety cue; C1–4, class 1–4.

For RT, there was no significant interaction effect (all, *p* >.05). However, a significant main effect of Condition suggests longer RT for CS+ compared to CS− [*F*(1,43) = 5.226, *p* =.027, *η^2^_p_* =.108]. Additionally, the main effect of Time indicates that participants judged faster in the late phase (128.36 ms) compared to the early phase (140.54 ms) [*F*(1,43) = 4.194, *p* =.047, *η^2^_p_* =.089]. No other significant main or interaction effect was found.

For SCL, there was a marginal main effect of Condition [*F*(1,44) = 3.445, *p* =.070, *η^2^_p_* =.073], which suggests a slightly larger SCL for CS+. Subsequent paired t-tests revealed that this marginal difference in SCL was observed in the early acquisition phase (*t* = 1.835, *p* =.073) but not in the late acquisition phase (*t* = 0.779, *p* =.440). No other significant main or interaction effects were found.

For HR, no significant main or interaction effect was found (all, *p* >.05).

For startle EMG, there was a marginal main effect of Condition [*F*(1,44) = 3.491, *p* =.068, *η^2^_p_* =.074], indicating a slightly larger startle response for CS+. Further examination through paired t-tests disclosed this marginal difference in the late acquisition phase (*t* = 1.946, *p* =.058) with no such difference observed in the early acquisition phase (*t* = 1.687, *p* =.099). No other significant main or interaction effects were found.

### 3.3 Generalization phase

**Figure 2:**
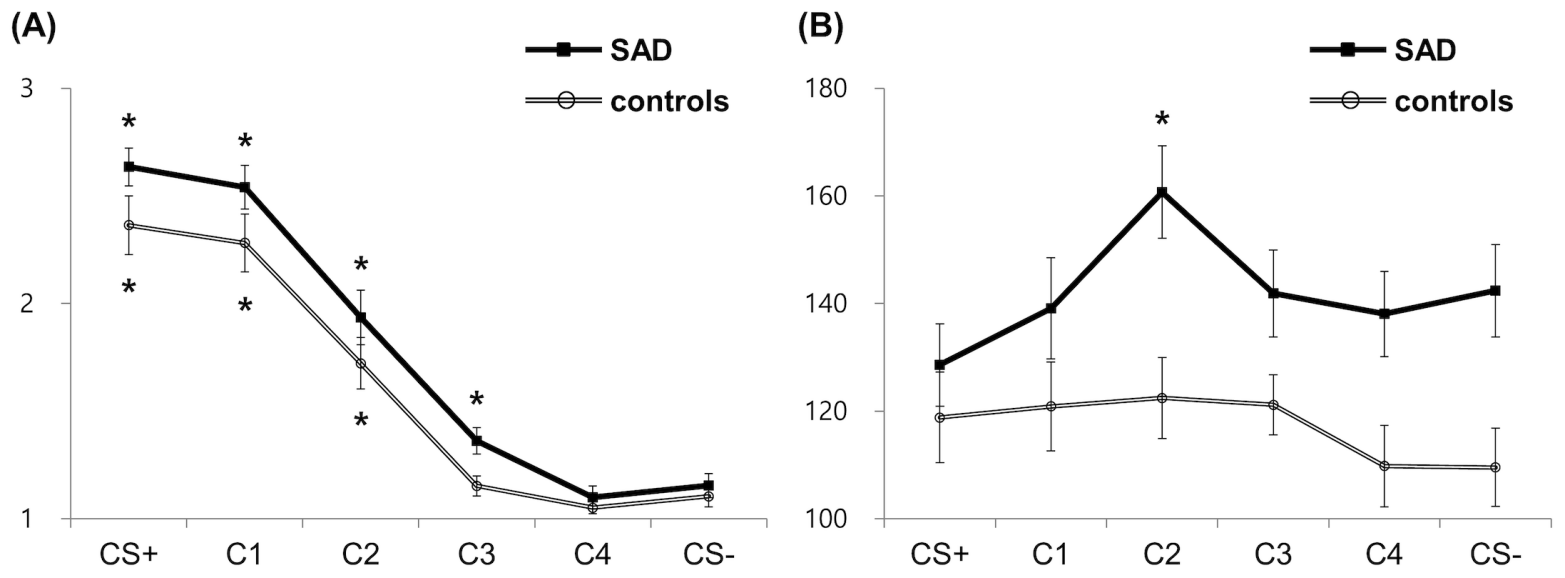
Behavioral results for risk rating **(A)** and response time **(B)**. Asterisks indicate significant differences from the reference condition (CS−) in each group (corrected *p* <.05). SAD, social anxiety disorder; CS+, threat cue; CS-, safety cue; C1-4, class 1-4.

In the generalized phase, the rmANOVA on risk rating revealed the significant main effects of Condition [*F*(2.035,95.650) = 157.811, *p* <.001, *η^2^_p_* =.771]. The rmANOVA on RT revealed the significant interaction effect [*F*(5,225) = 2.396, *p* =.038, *η^2^_p_* =.051] and main effects of Group [*F*(1,45) = 6.023, *p* =.018, *η^2^_p_* =.118] and Condition [*F*(5.225) = 4.770, *p* <.001, *η^2^_p_* =.096]. Further analyses on the interaction effect revealed a slower RT for C2, C3, C4, and CS− in patients compared to controls (**Figure 2B**), although the differences were marginal for C3 (CS+: *t* = −.733, *p* =.467; C1: *t* = −1.576, *p* =.122; C2: *t* = −3.149, *p* =.003; C3: *t* = −1.938, *p* =.059; C4: *t* = −2.403, *p* =.020, CS−: *t* = 2.90, *p* =.006), indicating patients’ difficulties in evaluating risk for both safe and ambiguous stimuli. To further examine the difference in stimuli generalization between SAD and control groups, subsequent planned comparisons between morphed stimuli (C1–4) and CS− were conducted for four pairs with the B–H correction in each group. The controls showed significantly higher ratings for C1 and C2 than CS− [for C1, mean difference (MD) = 1.22, *t*(22) = 8.537, *p* <.001; for C2, MD = 0.64, *t*(22) = 4.723, *p* <.001]. On the other hand, the patient group showed significantly higher ratings for C1–3 than CS− [for C1, MD = 1.40, *t*(25) = 11.915, *p* <.001; for C2, MD = 0.78, *t*(25) = 5.690, *p* <.001; for C3, MD = 0.21, *t*(25) = 3.065, *p* =.005], indicating that the patient group assessed the risk higher than CS− when the stimuli close to CS− contained a portion of the CS+ features (**Figure 2A**). While the controls showed comparable RTs in all classes of morphed stimuli compared to CS−, the patient group showed significantly slower RTs for C2 than CS− [MD = 18.31 ms, *t*(23) = 3.164, *p* =.004], indicating that the patient group took longer to assess the risk for ambiguous stimuli (**Figure 2B**).

**Figure 3:**
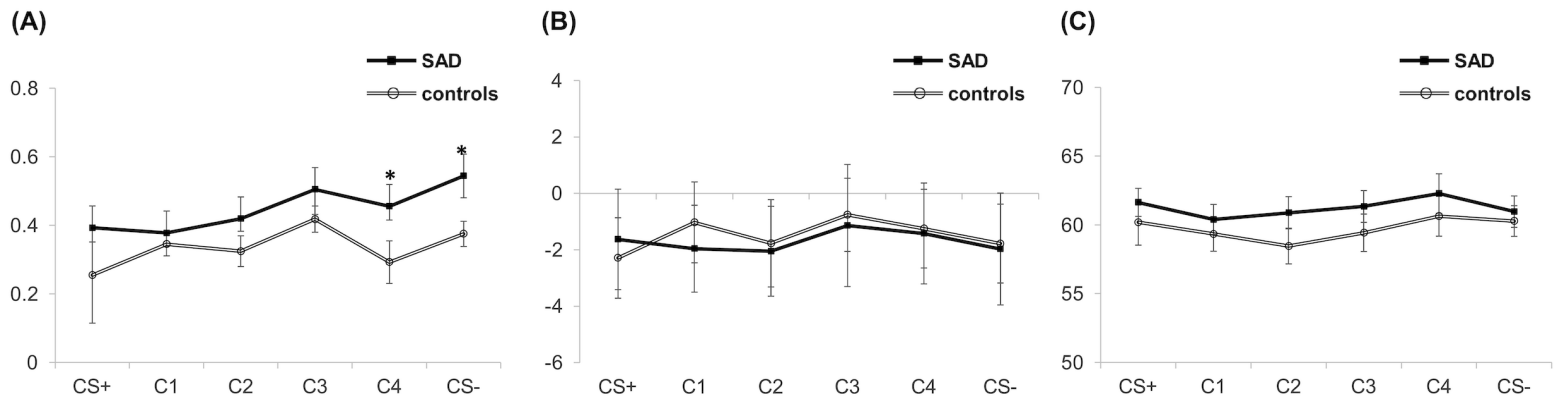
Physiological responses including SCL **(A)**, HR **(B)**, and startle EMG **(C)** during the generalization. Asterisks indicate significant differences between the two groups. (corrected *p* < .05). SAD, social anxiety disorder; CS+, threat cue; CS-, safety cue; C1-4, class 1-4; SCL: skin conductance level; HR: heart rate; EMG: electromyography.

The ANOVA on SCL, HR, and EMG yielded no significant main or interaction effect (all, *p* >.05) (**Figure 3**). For the SCL, although the effect of Condition falls short of significance [*F*(2.815,112.598) = 2.176, *p* = .099, *η^2^p* =.052], there was an observable tendency of higher SCL in response to safe signals (i.e., C4 and CS−) in patients (**Figure 3A**). Subsequent independent *t*-tests on group difference showed that patients exhibited significantly higher SCL compared to controls for C4 [MD = −0.17, *t*(42) = −2.211, *p* =.033] and marginally higher SCL compared to controls for CS− [MD = −0.15, *t*(40) = −1.845, *p* =.073].

### 3.4 Changes across phase

**Figure 4:**
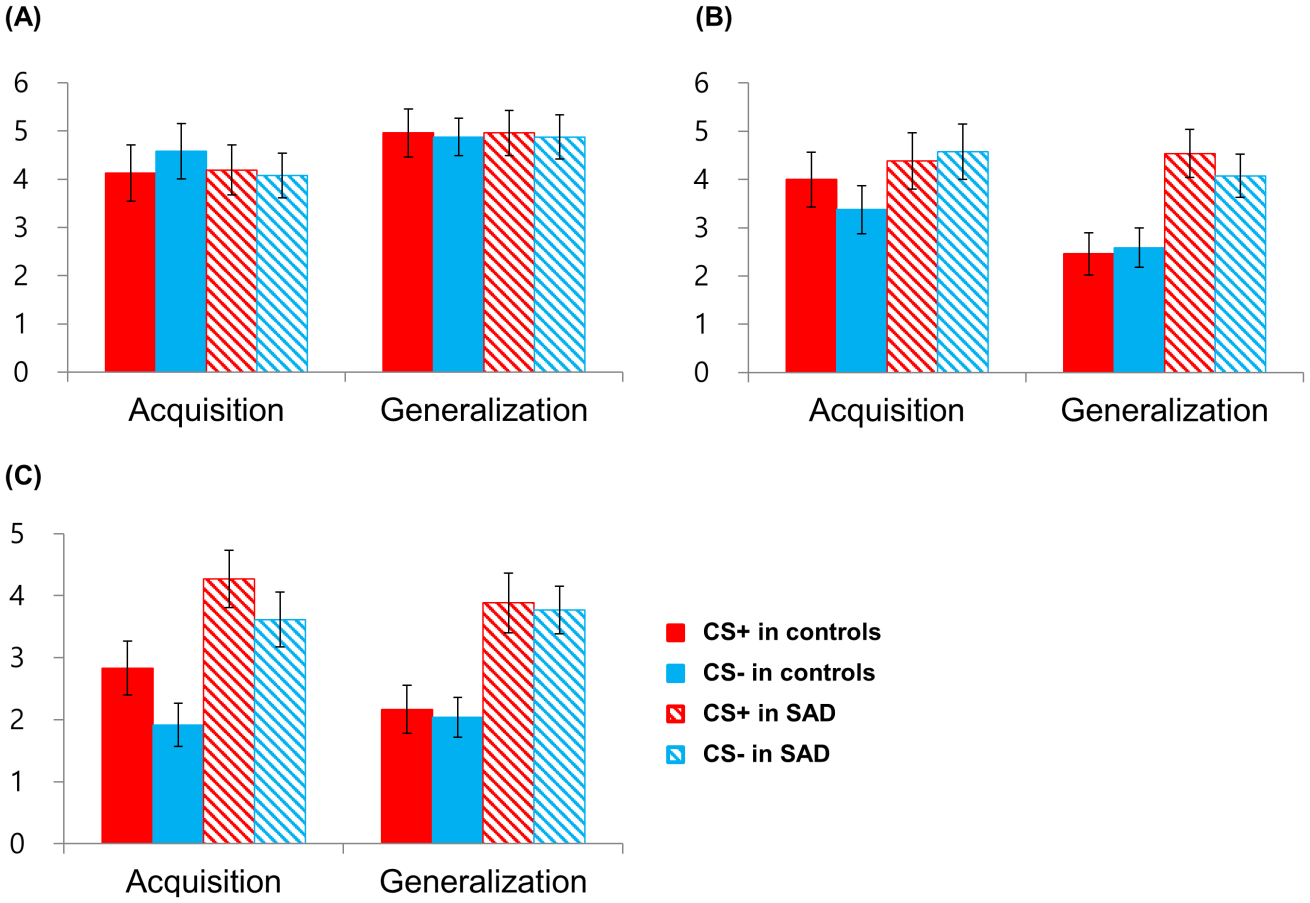
Retrospective subjective ratings of valence **(A)**, arousal **(B)**, and threat **(C)** for the threat (CS+) and the safety cue (CS−) after the acquisition and generalization phases. SAD, social anxiety disorder.

Subsequent analyses were conducted on subjective ratings of valence, arousal, and threat that were measured at the end of each phase (**Figure 4**). The rmANOVA on the valence rating revealed a significant main effect of Phase [*F*(1,47) = 4.129, *p* =.048, *η^2^_p_* =.081], participants rated the valence of CSs more negatively in the acquisition phase than in the generalization phase (**Figure 4A**). No other main or interaction effects were significant (all, 3*p* >.05). The rmANOVA on the arousal rating revealed the significant main effects of Group [*F*(1,47) = 7.809, *p* =.008, *η^2^_p_* =.142] and Phase [*F*(1,47) = 10.573, *p* =.002, *η^2^_p_* =.184] and the interaction effects of Group × Phase [*F*(1,47) = 5.964, *p* =.018, *η^2^_p_* =.113] and Group × Phase × Condition [F(1,47) = 4.260, p =.045, *η^2^_p_* =.083]. While controls showed lower arousal ratings for CSs in the generalization phase compared to the acquisition phase [CS+, *t*(22) = 3.869, *p* =.001; CS−, *t*(22) = 1.811 *p* =.084], patients did not show a decrease in the arousal rating in the generalization phase [CS+, *t*(25) = −.547, *p* =.589; CS−, *t*(25) = 1.247, *p* =.224], and the difference was particularly noticeable in CS+ (**Figure 4B**). This contrasting pattern shows normal habituation to CSs in controls but failed habituation in patients. The rmANOVA on the threat rating revealed the significant main effect of Group [*F*(1,47) = 15.431, *p* <.001, *η^2^_p_* =.247]; patients rated CSs as more threatening than controls across the acquisition and generalization phases (**Figure 4C**). The significant interaction effect of Phase × Condition [*F*(1,47) = 8.629, *p* =.005, *η^2^_p_* =.155] indicated that participants tended to rate CS+ as more threatening compared to CS− in the acquisition phase [*t*(48) = 1.823, *p* =.074], whereas ratings were comparable for CS+ and CS− in the generalization phase [*t*(48) =.343, *p* =.733].

We further investigated whether the observed pattern of habituation would also be evident in physiological dimension. The rmANOVA on EMG revealed the significant main effect of Phase [*F*(1,44) = 28.502, *p* <.001, *η^2^_p_* = .393]. In both groups, startle response decreased from the acquisition (for CS+: mean, 64.49; for CS−: mean, 63.57) to the generalization phase (for CS+: mean, 60.47; for CS−: mean, 60.66) regardless of Condition. The rmANOVA on SCL and HR showed no significant main or interaction effect (all, *p* >.05).

### 3.5 Correlations

In patients during the acquisition phase, risk ratings for CS− showed a significant correlation with SIAS (*r* = 0.420, *p* =.033) and SPS (*r* = 0.510, *p* =.008), a trend-level correlation with FNE (*r* = 0.369, *p* =.064), and a non-significant correlation with LSAS (*r* = 0.249, *p* =.220). In contrast, risk rating for CS+ was not associated with any of social anxiety scores (all, *p* >.05). In the generalization phase, no significant correlation was found in both behavioral and physiological outcomes (all, *p* >.05).

After controlling for trait anxiety, the correlation between risk ratings for CS− and social anxiety symptoms weakened, showing a significant correlation with SPS (*r* = 0.506, *p* = .019) and a trend-level correlation with SIAS (*r* = 0.417, *p* =.060), with no significant correlations with LSAS and FNE (all, *p* >.05). Notably, significant correlations emerged in the generalization phase, including associations between risk ratings for C4 and LSAS (*r* = 0.447, *p* = .042) and SPS (*r* = 0.446, *p* = .043), along with marginal correlations for CS- with LSAS (*r* = 0.417, *p* = .060) and SPS (*r* = 0.418, *p* = .059). Risk ratings for CS+ remained unassociated with any social anxiety scores (all, *p* >.05). In terms of physiological measures, the SCL for C4 significantly correlated with FNE (*r* = 0.495, *p* = .026), with no other significant correlations found (all, *p* >.05).

## 4 Discussion

Using an ecologically enhanced paradigm, we investigated whether patients with SAD differ from healthy controls in the processes of conditioned fear acquisition and generalization. During both the early and late acquisition phases, a higher risk rate for CS+ indicated that fear conditioning was successful irrespective of the group. While there was a trend for conditioning in a similar direction for SCL and startle EMG, it did not reach statistical significance. We found no group differences in risk rating, RT, physiological, or startle responses, except that patients reported higher subjective arousal levels and threat ratings than healthy controls. By contrast, group differences were apparent in the generalization phase. While healthy controls generalized their conditioned fear response (i.e., risk rating) from the CS+ to two generalization stimuli (C1 and C2), patients’ fear response was transferred further to C3, which is closer to CS− than CS+. In addition, for ambiguous and safe stimuli, patients spent a much longer time evaluating the risk and exhibited higher SCL compared to controls. Interestingly, patients with a high level of fear of negative evaluation showed larger SCL in response to C4. In addition, within-group differences were also observed in the subjective arousal levels between phases; while controls experienced a decrease in arousal levels from the acquisition to the generalization phase, patients consistently demonstrated heightened arousal throughout the study. Taken together, although there were no typical and robust patterns of overgeneralization of physiological and startle responses in patients in the present study, our results imply that SAD patients have lower thresholds for provoking a conditioned fear response and exhibit less habituation.

In line with previous studies (17, 41), we did not find evidence for enhanced conditionability in patients during acquisition. Our behavioral results, including RT and risk rating, imply heightened fear generalization in SAD patients. The observed patient-group differences in RT, i.e., slower RT for safe (but not for threat) stimuli in patients, are consistent with previous findings that anxiety patients and controls show different fear responses to CS−, but not to CS+ [for a review, see (9)]. Greater fear responses to CS− have been interpreted as reflecting fear overgeneralization or impaired fear inhibition in patients (9). Notably, within patients, higher risk ratings for safe and ambiguous stimuli were positively correlated with social anxiety symptoms after controlling for trait anxiety. Specifically, higher ratings for C4 positively correlated with the LSAS and SPS, while ratings for CS− showed marginal positive correlations. In addition, patients with a higher level of fear of negative evaluation exhibited significantly larger SCL for C4. Inter-individual differences including trait anxiety have been intensively investigated in relation to fear learning (42), and trait anxiety has been reported to influence fear generalization in healthy controls (43). Our results indicate that even after controlling for these inter-individual differences, there were still significant associations between the level of social anxiety and overgeneralization for social threat. Together with risk rating results, our RT data suggest that patients have difficulties in evaluating the risks of safe and ambiguous signals in an aversive context, implying an associative learning deficit in patients with SAD. In addition, within-group differences in subjective reporting across phases also suggest maladaptive non-associative learning in SAD. While controls showed a decrease in subjective arousal from the acquisition to the generalization phase, patients’ arousal state was consistently higher throughout the two phases. According to the theoretical framework, non-associative learning includes persistent or increasing fear reactivity to novel stimuli, which derives from pathological failure to habituate or sensitization (44). To summarize, SAD patients experience difficulties distinguishing between threat and safety cues and adapting to novel stimuli. We assume that failure to habituate makes patients persistently anxious during an aversive context, making fear extinction more difficult.

While the behavioral dimension (i.e., risk rating and RT) showed a significant group difference in fear generalization, physiological responses were comparable between the two groups. The lack of physiological discrimination between CS+ and CS− in the generalization phase may be explained separately in controls and SAD. For the controls, we observed a general decrease in both subjective arousal and fear-potentiated startle responses across phases, suggesting a habituation effect despite occasional presentations of the US in the generalization phase. This overall reduction in physiological reactivity likely contributed to the diminished discrimination between CS+ and CS−. In contrast, the patient group maintained high levels of subjective arousal from the acquisition to the generalization phase, indicating a lack of habituation. Notably, patients exhibited elevated SCL to CS− compared to controls in the generalization phase. This heightened response to the safety cue might have obscured the physiological discrimination between CS+ and CS−. The dissociation between behavioral and physiological responses in fear conditioning has been reported in a previous lesion study (45). Bechara et al. demonstrated that declarative associative learning, represented by risk rating, is impaired in patients with bilateral hippocampal damage, whereas implicit learning, as reflected by autonomic nervous system responses, is compromised in patients with bilateral amygdala damage (45). This suggests the possibility that potentially distinct neural mechanisms may be involved in implicit and explicit learning. Similarly, dissociation between self-reported arousal/anxiety and the physiological response to emotional stimuli has often been reported in studies of clinical and subclinical samples of anxiety (46–50). For example, socially anxious individuals reported feeling more anxious during public speaking compared to controls despite equal patterns of physiological arousal, which suggests that increased awareness of or sensitivity to arousal, rather than the level of physiological arousal per se, may be a more important factor in characterizing SAD. One possible explanation for similar phenomena in this study is that socially relevant US was aversive enough to elicit fear conditioning as defined by cognitive awareness and arousal but not sufficient to generate a physiological response, as is often seen in human fear conditioning paradigms. It has been suggested that studies using a startle probe should use a more highly aversive US (19, 51, 52).

Alternatively, some have attempted to attribute this discrepancy to the idiosyncrasies of the socially anxious sample. When they encounter a social threat, they presumably show avoidance and submissiveness rather than a fight-or-flight response and thus exhibit less autonomic activation (48). In addition, inward-directed attention (which makes an external startle probe less effective) and a high comorbidity rate with major depression in social anxiety are also considered major causes of the lack of physiological activation (53, 54). Previous studies on fear conditioning in social anxiety have revealed nonsignificant or weak physiological evidence of fear (over)generalization (17, 41) leading to the conclusion that social anxiety may not be characterized by strong fear (over)generalization. On the other hand, a recent meta-analysis (14) and the Hierarchical Taxonomy of Psychopathology (HiTOP) model (55) advocate for a transdiagnostic approach in understanding fear generalization. The meta-analysis revealed heightened fear generalization across anxiety-related disorders, emphasizing its transdiagnostic nature. Similarly, the HiTOP model proposes a dimensional framework that transcends traditional taxonomies, providing a cross-cutting perspective on psychopathological syndromes. Both studies highlight the importance of adopting a unified view, moving beyond disorder-specific boundaries to better comprehend fear-related phenomena like generalization across various anxiety-related disorders. In this context, intolerance of uncertainty (IU), a transdiagnostic construct encompassing both personality and cognitive biases that assesses the inclination to perceive uncertainty as aversive, has emerged as a crucial factor in classical threat conditioning mechanisms. It has been found that a high level of self-reported IU, over trait anxiety and worry, was related to greater generalization (42, 56, 57). As we have not measured subjective IU or manipulated outcome uncertainty as in previous studies (58), further discussion on these aspects is not feasible within the scope of our research. However, it appears crucial to incorporate uncertainty-related factors in future fear learning studies, considering the potential significance of such elements.

The primary strength of this study is that we employed ecologically enhanced social-anxiety-related stimuli, such as angry faces and contemptuous comments. Given the prior neuroimaging studies reporting that directly facing angry and contemptuous facial expressions provokes strong neural responses in participants with SAD (59, 60), our study design seemed suitable for investigating fear acquisition and generalization in SAD patients who are sensitive to others’ evaluations. However, it is important to note that the use of disorder-relevant US generally offers both methodological advantages and challenges (61). On the one hand, this approach enhances ecological validity by providing conditions more closely aligned with the real-world experiences of patients with SAD. Such an experimental design may facilitate the capture of more clinically relevant behavioral and physiological patterns, potentially yielding deeper insights into the mechanisms of fear acquisition and generalization in patients. On the other hand, although angry faces or verbal rejection are a social threat signal eliciting fear responses in healthy adults (20, 62), the inherent aversiveness of the US may potentially vary between patients with SAD and healthy controls (59, 60). This difference makes it challenging to discern whether group differences in fear responses stem from variations in fear acquisition and generalization processes or from disparities in the perceived aversiveness of the stimuli themselves. Although these two factors cannot be disentangled in the present study, future studies could use both disorder-relevant and irrelevant USs to evaluate their differential effects in clinical populations. Furthermore, future research could benefit from the inclusion of diverse comparison groups. In this study, the use of psychiatrically healthy adults as a control group might not have been optimal for fully capturing the effects of employing disorder-specific stimuli. A more relevant control group might consist of individuals in recovery from SAD or subclinical populations that are likely to have a similar baseline sensitivity to SAD-specific stimuli. This shared characteristic could provide a more sophisticated understanding of the differences in fear acquisition and generalization specifically attributable to current SAD symptoms, rather than to differences in the perceived relevance or salience of the stimuli.

The present study had several limitations. First, since our power calculation was based on a previously reported effect size of fear acquisition, the sample size of 25 per group might not be sufficient to robustly capture the interaction between the stimulus type and group during the generalization phase. Second, the self-reported risk rating that we used to evaluate participants’ perceived likelihood of a person on the screen making contemptuous comments consisted of a simple 1–3 Likert scale, which was too simple to reveal the variability of the fear response. The use of a three-point Likert scale can limit the ability to analyze the results of a measurement. As it only categorizes three possible responses to the risk rating, it is difficult to get information about the intensity or degree of the response. When a subject selects “2,” the interpretation can be ambiguous because it can have multiple meanings. Therefore, it may not accurately reflect the risk expectancy of the subjects, which limits the interpretation of the results of this study. Third, it should be noted that the present study recruited only an Asian population, which has a low prevalence of anxiety disorders presumably due to cultural characteristics such as a high level of emotional control (63). For example, a previous study investigating the effect of ethnicity on startle response showed that Asians demonstrate less emotional expression (64), smaller fear-potentiated startle response in the baseline period (65), and reduced sensitivity to unpredictable threats (66). This emphasizes that biomarkers of emotional response should be interpreted with caution. Consequently, the consideration of challenges and biases in recording electrodermal activity in different ethnicities underscores the critical need for a more inclusive approach in psychophysiological research (67). In this vein, the exclusive recruitment of Asian participants can be seen as a strength in the current study. Many research studies predominantly feature participants from Western populations, leading to a gap in cross-cultural representation. By focusing on Asian participants, our research contributes to a more comprehensive understanding of fear generalization across diverse cultural contexts. Finally, we did not measure participants’ subjective rating for startle stimuli. Due to the possibility that the startle probe could make participants feel CS aversive as well, it is recommended that studies using startle probes include a larger number of trials or a rating for startle probes (68).

## 5 Conclusion

This study examined whether an ecologically enhanced fear conditioning paradigm using more disorder-specific stimuli can elicit a distinct pattern of overgeneralization in patients with SAD. Using social-anxiety-related stimuli such as an angry face and contemptuous comments, we found that SAD patients assessed the risk higher for ambiguous stimuli, spent more time than healthy controls when evaluating safe and ambiguous stimuli, and showed consistently high levels of subjective arousal. Although we did not find a typical and robust pattern of overgeneralization of physiological and startle responses in patients, our results imply that SAD patients have lower thresholds for provoking a conditioned fear response, making it more difficult for them to discriminate between threat and safety cues.

## Data Availability

The raw data supporting the conclusions of this article will be made available by the authors, without undue reservation.
