# Supplementary material for: Overgeneralization of conditioned fear in patients with social anxiety disorder
Source: Front Psychiatry. 2024 Aug 23;15:1415135. doi: 10.3389/fpsyt.2024.1415135 (PMC11378195; doi:10.3389/fpsyt.2024.1415135)
Supplement: Supplementary file 1 [file Table1.docx]

**Supplementary Material for**

**Overgeneralization of conditioned fear in patients with social anxiety disorder**

**Unconditioned Stimulus Selection Details**

Separate from the main experiment, ten respondents (male participants, N = 3, 30%) who did not participate in the main experiments were recruited from the community to assess neutral and contemptuous comment that would serve as an unconditioned stimulus. A total of 27 contemptuous and 18 neutral comments was provided, and respondents had to rate valence (from negative to positive), arousal (from low to high), and threat (from not at all to threatening) levels for those comments in a 9-point Likert scale. Based on these scales, ten items were selected for each stimulus set (e.g., 'banana is yellow' for neutral stimulus and 'you are good for nothing!' for negative stimulus). The skewness was reasonably balanced across the ratings except for arousal of negative contents, which implicates the well-characterized stimuli set used in the study.

**Supplementary Table 1. Pre-experiment ratings on the content of the audio stimuli**

| **Variable** | **Neutral content (N=10)** | | | ***skp*** | **Contemptuous content (N=10)** | | | | ***skp*** |
| --- | --- | --- | --- | --- | --- | --- | --- | --- | --- |
|  | mean | SD |  | | | mean | SD |  | |
| Valence rate, 1-9 | 4.93 | 0.29 | .849 | | | 1.48 | 0.11 | .478 | |
| Arousal rate, 1-9 | 1.48 | 0.31 | -.265 | | | 7.55 | 0.18 | -1.97 | |
| Threat rate, 1-9 | 1.34 | 0.23 | -.646 | | | 6.54 | 0.43 | .359 | |

*Skp*, Skewness of data; SD, standard deviation.

**Supplementary Table 2.** Repeated measures ANOVA of skin conductance level with different time windows

|  | **F** | | | |
| --- | --- | --- | --- | --- |
| **Effect** | **0.5–6** | **1–6 (original)** | **1.5–6** | **1-4** |
| **Acquisition** |  |  |  |  |
| Main effect |  |  |  |  |
| Group (controls vs SAD) | **4.350^*^** | 0.209 | 0.547 | 0.589 |
| Condition (CS+ vs CS-) | 1.152 | **3.445^†^** | **3.403^†^** | **3.373^†^** |
| Phase (early vs late) | 1.055 | 2.650 | 1.795 | **3.944^*^** |
| Interaction effect |  |  |  |  |
| Group x Condition | 1.318 | 0.784 | 0.052 | 0.074 |
| Group x Phase | 1.651 | 0.372 | 0.604 | 0.354 |
| Phase x Condition | 0.770 | 1.516 | 0.499 | 2.462 |
| Group x Condition x Phase | 1.441 | 0.222 | 1.161 | 0.715 |
| **Generalization** |  |  |  |  |
| Main effect |  |  |  |  |
| Group (controls vs SAD) | **4.270^*^** | **3.129^†^** | **5.647^*^** | **6.685^*^** |
| Condition (CS+ vs CS-) | 0.880 | 2.176 | 1.459 | 1.779 |
| Interaction effect |  |  |  |  |
| Group x Condition | 1.047 | 0.510 | 0.303 | 0.341 |

SAD, social anxiety disorder; CS+, threat signal; CS-, safety signal.

^*^, *p* < .05

^†^, *p* < .09

**Supplementary Table 3.** Repeated measures ANOVA of behavioral and physiological measures with Beck Depression and Anxiety Inventory (BDI and BAI) scores as covariates

| **Effect** | **F** | ***p* value** | **Effect size** |
| --- | --- | --- | --- |
| **Early Acquisition** |  |  |  |
| Risk rating |  |  |  |
| Group (controls vs SAD) | > .001 | .986 | > .001 |
| Condition (CS+ vs CS-) | 49.583 | **< .001** | .553 |
| 2-Group x 2-Condition | 1.624 | .210 | .039 |
| RT |  |  |  |
| Group (controls vs SAD) | 1.695 | .201 | .042 |
| Condition (CS+ vs CS-) | 3.496 | .069 | .082 |
| 2-Group x 2-Condition | 0.118 | .733 | .003 |
| SCL |  |  |  |
| Group (controls vs SAD) | 1.047 | .313 | .028 |
| Condition (CS+ vs CS-) | 0.305 | .584 | .008 |
| 2-Group x 2-Condition | 0.107 | .745 | .003 |
| HR |  |  |  |
| Group (controls vs SAD) | 0.429 | .517 | .012 |
| Condition (CS+ vs CS-) | 0.038 | .846 | .001 |
| 2-Group x 2-Condition | 0.550 | .463 | .015 |
| Startle EMG |  |  |  |
| Group (controls vs SAD) | 0.121 | .730 | .003 |
| Condition (CS+ vs CS-) | 4.705 | **.036** | .110 |
| 2-Group x 2-Condition | 0.505 | .482 | .013 |
| **Late Acquisition** |  |  |  |
| Risk rating |  |  |  |
| Group (controls vs SAD) | 0.098 | .756 | .003 |
| Condition (CS+ vs CS-) | 89.897 | **< .001** | .697 |
| 2-Group x 2-Condition | 0.053 | .819 | .001 |
| RT |  |  |  |
| Group (controls vs SAD) |  |  |  |
| Condition (CS+ vs CS-) | 0.116 | .736 | .003 |
| 2-Group x 2-Condition | 2.937 | .095 | .074 |
| SCL |  |  |  |
| Group (controls vs SAD) | 0.141 | .709 | .004 |
| Condition (CS+ vs CS-) | 0.646 | .427 | .018 |
| 2-Group x 2-Condition | 0.073 | .788 | .002 |
| HR |  |  |  |
| Group (controls vs SAD) | 2.021 | .164 | .055 |
| Condition (CS+ vs CS-) | 2.259 | .142 | .061 |
| 2-Group x 2-Condition | 0.581 | .451 | .016 |
| 2-Group x 2-Condition | 0.214 | .647 | .006 |
| Startle EMG |  |  |  |
| Group (controls vs SAD) | 0.132 | .718 | .003 |
| Condition (CS+ vs CS-) | 6.597 | **.014** | .148 |
| 2-Group x 2-Condition | 0.214 | .647 | .006 |
| **Generalization** |  |  |  |
| Risk rating |  |  |  |
| Group (controls vs SAD) | 1.309 | .259 | .032 |
| Conditions (CS+, C1-C4, CS-) | 48.704 | **< .001** | .549 |
| 2-Group x 6-Condition | 1.552 | .175 | .037 |
| RT |  |  |  |
| Group (controls vs SAD) | 5.965 | **.019** | .133 |
| Conditions (CS+, C1-C4, CS-) | 3.787 | **.003** | .089 |
| 2-Group x 6-Condition | 3.633 | **.004** | .085 |
| SCL |  |  |  |
| Group (controls vs SAD) | 4.515 | **.041** | .114 |
| Conditions (CS+, C1-C4, CS-) | 0.331 | .894 | .009 |
| 2-Group x 6-Condition | 0.358 | .877 | .010 |
| HR |  |  |  |
| Group (controls vs SAD) | 0.049 | .825 | .001 |
| Conditions (CS+, C1-C4, CS-) | 2.211 | .078 | .263 |
| 2-Group x 6-Condition | 0.685 | .638 | .099 |
| Startle EMG |  |  |  |
| Group (controls vs SAD) | 0.359 | .553 | .009 |
| Conditions (CS+, C1-C4, CS-) | 1.088 | .368 | .028 |
| 2-Group x 6-Condition | 0.475 | .795 | .012 |

SAD, social anxiety disorder; RT, reaction time; SCL, skin conductance level; HR, heart rate; EMG, electromyography; CS+, threat signal; CS-, safety signal; C1-4, class 1-4.

Bold indicates statistical significance (*p* < .05).
